# Accurate measurement of sulfhydryls and TCEP-releasable sulfhydryls in the liquid phase of wine that contribute to ‘reductive’ aromas using LC-MS/MS

**DOI:** 10.1016/j.heliyon.2024.e28929

**Published:** 2024-04-01

**Authors:** Marlize Z. Bekker, Maryam Taraji, Vilma Hysenaj, Natoiya Lloyd

**Affiliations:** a The Australian Wine Research Institute, P.O. Box 197, Glen Osmond, SA, 5064, Australia; b School of Agriculture and Food Sustainability, The University of Queensland, St Lucia, QLD, 4067, Australia; c Metabolomics Australia, PO Box 197, Adelaide, SA 5064, Australia

**Keywords:** Sulfhydryls, Disulfides, Polysulfides, Hydrogen sulfide, Ferrocene-based maleimides, TCEP, Precursors

## Abstract

Volatile sulfur compounds (VSCs) are important aroma and flavour characters in food and beverage products. The identification and quantification of these extremely reactive and volatile compounds pose analytical challenges which demand selective and sensitive methods. In this study, a novel quantification method was developed to analyse sulfhydryls as well as the total pool of sulfhydryls which can be released after tris(2-carboxyethyl)phosphine (TCEP) addition from disulfides, polysulfides, metal-bound and other yet to be identified sources naturally present in wine. The majority of methods for VSC quantification analyse VSCs in wine headspace, whereas this method measures sulfhydryls and TCEP-releasable sulfhydryl species, which likely include free and metal-bound sulfhydryl forms, in the liquid phase of wine using UHPLC-MS/MS. Sulfhydryls were derivatised with N-(2-ferroceneethyl) maleimide **(**FEM**)**, subsequently, followed by differential labelling of sulfhydryls released after TCEP addition with ferrocenecarboxylic acid-(2-maleimidoyl)ethylamide (FMEA). Analysis of commercial wines revealed the presence of hydrogen sulfide, methanethiol, ethanethiol, and 2-mercaptoethanol at aroma-active concentrations. Significant positive correlations were found between MeSH and CH_3_–S-R TCEP-releasable species, and significant positive correlations were found between EtSH and CH_3_–CH_2_–S-R TCEP-releasable species. This method provides important information on sulfhydryls, and may also provide insights into a wine's risk of developing ‘reductive’ faults post-bottling from latent sources.

## Highlights

• Highly selective and sensitive method that quantifies sulfhydryls in wine.
• Quantification of the total pool of TCEP-releasable sulfhydryls in wine.
• Provides insights into the pool of latent sources of ‘reductive’ aromas.
• Significant positive correlations between sulfhydryls and TCEP-releasable species.

## 1 Introduction

Volatile sulfur compounds (VSCs) are important for modulating the aroma and flavour of a broad range of food and beverage products. Certain VSC species, such as hydrogen sulfide (H_2_S) and methanethiol (MeSH), belong to the most potent aroma substances that are known in the food and beverage industry, and trace amounts of these compounds negatively impact aroma and flavour as well as the perceived quality of food and beverage products. Volatile sulfur compounds are particularly important for imparting unwanted aromas in wine, which have significantly negative effects on the perceived quality of wine and consumer preference [1]. These sulfidic off-aromas are commonly referred to as ‘reductive’ aromas, and they can described as ‘rotten egg’, ‘sewage’, ‘cooked vegetables’ and ‘rotten cabbage’ [1,2]. In fact, ‘reductive’ aroma faults imparted by sulfur compounds are one of the main wine faults and it affects a large portion of wines produced in all wine-producing regions of the world [3].

The formation of the VSCs is driven by yeast metabolic activity and chemical changes during fermentation, with most of the undesirable VSCs produced by yeast [4]. The compounds most commonly associated with the undesirable sensory perception of ‘reductive’ aromas are H_2_S, MeSH, and ethanethiol (EtSH) [5,6]. These sulfur compounds are reactive nucleophiles and they readily react with other wine compounds, such as tannins, polyphenols, and metals, or simply autoxidise with other sulfhydryl species. Therefore they can be present as free compounds, loosely-bound to metals such as copper in wine [7], or irreversibly bound as quinone-sulfhydryl adducts with VSCs [8]. Certain of the larger molecular weight sulfhydryl compounds (*e.g.* disulfides, polysulfides, metal-bound complexes) may release sulfhydryls during wine storage under the right conditions [6,9,10].

The processes responsible for the liberation of sulfhydryls and reformation of larger molecular weight sulfur compounds are not completely understood [11]. Some of the processes responsible for the liberation of sulfhydryls include sulfitolysis, thiosulfatolysis, and thiolysis [1,9,11,12]. These processes may all play important roles in modulating the liberation of H_2_S and MeSH from the pool of precursors present in wine. The addition of high halide concentrations (for metal-bound forms) or strong reducing agents such as tris(2-carboxyethyl)phosphine (TCEP) (for disulfides, polysulfides, and certain other bound forms) to wine also facilitates the conversion of the latent sources into free sulfhydryls [9]. As such, gaining an understanding of the amount of sulfhydryls present in a wine, as well as the total pool of disulfides, polysulfide, and other sulfur compounds that have the potential to act as latent sources for sulfhydryl release post-bottling, is important to accurately gauge the potential of a wine to display ‘reductive’ aromas post-bottling.

Volatile sulfur compounds are not only responsible for negative aromas, in fact, certain highly sought-after and important varietal and specific wine-style aromas are also imparted by sulfur compounds. For example, 3-sulfhydrylhexan-1-ol (3SH) and phenylmethanethiol (PMT) play an important role in modulating wine aroma, both in terms of imparting positive ‘tropical’ and ‘gun flint’ aromas associated with certain wine varieties and wine styles. PMT in particular is an important compound when considering ‘reductive’ aromas, seeing that winemakers anecdotally refer to PMT as “positive reduction”, “struck flint” or “minerality” and this aroma character is highly sought after in certain styles of Chardonnay [13,14]. Considering that the presence of PMT, especially at high concentrations, may be mistaken for burnt rubber aroma or struck match [15], it is important to also consider this compound when quantifying VSCs in wine.

The identification and quantification of these extremely reactive and volatile compounds pose analytical challenges which demand selective and sensitive methods which are significantly impacted by the diverse and often complex food or beverage matrices. Various methods are available for the detection of VSCs. Considering that these compounds are highly volatile and contribute to wine aroma rather than wine flavour, gas chromatography (GC) is most commonly used to determine VSC concentrations in the headspace of wines. The most common detection systems coupled to GC are sulfur chemiluminescence detection (SCD), flame photometric detection (FPD), and mass spectrometry (MS) [7,16,17]. Liquid chromatography-mass spectrometry (LC-MS) methods are not generally implemented for the analysis of ‘reductive’ VSCs associated with ‘reductive’ aromas, due to the volatility of the compounds and the simple chemical structure of the compounds [18]. Direct headspace injection is the most commonly used sample introduction method for the headspace analysis of wine samples [17,19,20]. In certain instances, solid phase micro-extraction (SPME) is also used for the headspace analysis of VSCs [16,21]. There are a few reports of the derivatisation of sulfur compounds associated with ‘reductive’ aromas as well as ‘flint’ aromas prior to GC-MS analysis [22,23]. Sulfur compounds associated with more positive aroma characters, namely 3SH, 3SHA, and PMT, are commonly analysed using methods utilising a derivatisation step prior to analysis and also analysed using LC-MS [24].

When considering the total pool of sulfhydryl compounds in wine, the group that has been most elusive to quantification includes the putative precursors responsible for the release of VSCs during wine ageing. Many methods have been developed to address this knowledge gap, and all have had success in quantifying specific precursor moieties, however, the diversity in putative precursor makes it challenging to quantify the total precursor pool using one method. There are excellent examples of methods that target polysulfides as well as sulfur-containing amino-acid species [[25], [26], [27]]. Chen et al. [28] first described an approach using TCEP to release VSCs from copper sulfide complexes, and Kreitman et al. [26] quantified total bound and free H_2_S, MeSH, and EtSH in wines also utilising an approach consisting of TCEP addition to wine in combination with the addition of a copper (I) chelating agent (bathocuproinedisulfonic acid, BCDA) and cysteine. Ferreira et al. [11] also described a method based on the liberation of VSCs from their precursors using TCEP, and in this method, the liberated sulfhydryls that were produced by the TCEP reduction step were captured through complexation of these sulfhydryl compounds in copper (I) solutions. Using this release and trapping technique, Ferreira's group demonstrated that up to 400 μg/L of H_2_S and 58 μg/L of MeSH can be released in 68 days from wine samples.

It is clear that the quantification of VSCs in wine is complex with many factors contributing to the total concentration measured in different wine matrices (headspace, liquid phase, including metal and/or chelator additions). In this study, our aim was to develop a method to quantify and distinguish between sulfhydryls and TCEP-releasable sulfhydryls present in the liquid phase of a wine.

## 2 Materials and methods

### 2.1 Chemicals and reagents

Water was purified using a Milli-Q water purification system (Millipore, North Ryde, NSW, Australia). HPLC-grade acetonitrile (ACN) was purchased from Merck (Frenchs Forest, NSW, Australia). Ethanol (EtOH, 99.5 %) and formic acid (98 %) were purchased from Rowe Scientific Pty Ltd (Lonsdale, SA, Australia). 4-Acetaminothiophenol (AATP, 90 %), acetic acid glacial (100 %), ammonium bicarbonate (99.5 %), ammonium formate (10 M solution), anisaldehyde (98 %), butylthiol (BuSH, 99 %), *tert*-butylthiol (*t*-BuSH, 99 %), diethyl ether (99.3 %), dimethyl disulfide (DMDS, 99 %), diethyl disulfide (DEDS, 99 *%*), dimethylformamide (DMF, 99.9 %), ethanethiol (EtSH, 99.7 %), ethylenediamine tetraacetic acid (EDTA), hydrochloric acid (37 %), lithium aluminium hydride (95 %), oxalyl chloride (99.99 %), 1-pentanethiol (98 %), *iso-*propanethiol (*i*-PrSH, 97 %), phenylmethanethiol (PMT, 99.8 %), propanethiol (PrSH, 97 %)*,* 2-mercaptoethanol (2 ME, 99 %), tris-(2-carboxyethyl)-phosphine hydrochloride (TCEP), sodium hydroxide (99.99 %), sodium sulfate (anhydrous, 99 %), sodium sulfide nonahydrate (Na_2_S·H_2_O, 98 %), sodium thiomethoxide (NaSMe, 95 %), tetrahydrofuran (THF, 99.9 %), triethylamine (99 %), thiourea (99 %), and urea (99.5 %) were obtained from Sigma-Aldrich (Castle Hill, NSW, Australia). Ethane-*d5*-thiol (*d5*-EtSH, 99.5 %), *d5*-phenylmethanethiol (*d5*-PMT, 99.6 %) and *d3*-dimethyl disulfide (*d6*-DMDS, 99.5 %) were purchased from CDN Isotopes (Pointe-Claire, Quebec, Canada). Aluminium chloride (anhydrous, 99.99 %), 2-ferroceneacetonitrile (97 %), ferrocene carboxylic acid (98 %), and magnesium sulfate anhydrous (99.5 %) were purchased from Alfa Aesar. Dichloromethane (99.7 %), ferrocenoyl chloride (96 %), and methanol (99.9 %) were purchased from Merck.

### 2.2 Synthesis of derivatising reagents

Two derivatisation agents, N-(2-ferroceneethyl) maleimide **(**FEM**)** and ferrocenecarboxylic acid-(2-maleimidoyl)ethylamide (FMEA) were synthesized as described by Seiwert and Karst [29,30]. A detailed description of the synthesis is provided in Supporting Information (Supporting Information Section S1; Figures S1, S2).

### 2.3 Reference compound solutions

Two working solutions containing a mixture of PrSH, iPrSH, BuSH, EtSH, 2 ME, PMT, DMDS, and DEDS (1 mg/L and 0.1 mg/L of each compound in the respective working solutions) were prepared in a cold (4 °C) buffer solution (Buffer A: 12 % EtOH and 1 mM EDTA in 0.05 % formic acid in milli-Q-water). These solutions were stored at – 20 °C and used for up to 6 months. Two working solutions containing a mixture of H_2_S and MeSH (1 mg/L and 0.1 mg/L of each compound in the respective working solutions) were prepared in cold (4 °C) Buffer A. These solutions were prepared fresh daily and discarded after use. The working solutions were used to prepare standard solutions containing 10 VSCs (0.0, 0.1, 2.0, 0.5, 10.0, 25.0, 50.0, 100.0, and 200.0 μg/L for H_2_S, MeSH, EtSH, PrSH, iPrSH, BuSH, 2 ME, PMT, DMDS, and DEDS) using cold (4 °C) Buffer A.

An aqueous solution containing a mixture of deuterated *d6*-DMDS (0.6 mg/L), *d5*-EtSH (0.6 mg/L), and *d5*-PMT (0.6 mg/L) were prepared in the same buffer and used as internal standards. 10 μL of deuterated internal standards was added to the samples before the derivatisation which gives a final concentration of 50 μg/L for each of the deuterated standard.

### 2.4 Derivatisation procedure for samples and calibration standards

All reagents, standard solutions, and samples were kept cold (4 °C) throughout the derivatisation procedure.

FEM (30 μL, 5 mM in ACN) and ammonium bicarbonate (1 M, 30 μL) were added to amber vials (2 mL, Agilent Technologies, Forest Hill, Australia). Vials were capped and vortexed for 15 s and left for 1 min. Wine samples (120 μL) were added to vials, the samples vortexed for 15 s, and deuterated internal standards (10 μL, 0.6 mg/L) were added to the samples. Samples were then vortexed for 15 s, left at room temperature (20 °C) and vortexed every 5 min for a total of 15 min to allow the derivatisation process to complete. Urea (25 μL, 40 mM in ACN) was then added and the sample vortexed for 15 s, and then left for 1 min. TCEP (20 μL, 25 mM in 10 mM ammonium bicarbonate solution) was then added and the sample vortexed for 15 s every 5 min for a total of 15 min. FMEA (60 μL, 20 mM in ACN) was then added and the samples vortexed for 15 s every 5 min for a total of 15 min. After 15 min the derivatisation was complete and urea was added (75 μL, 40 mM in ACN), followed by formic acid (20 μL, 25 %) and ascorbic acid (30 μL, 100 mM, 10 % (v/v) formic acid). Samples (300 μL) were then transferred to amber vials containing a glass micro insert (250 μL, Agilent Technologies, Forest Hill, Australia) and analysed using liquid chromatography-mass spectrometry (LC-MS/MS).

### 2.5 UHPLC-MS/MS analysis

An Agilent 1290 Infinity ultra-high performance liquid chromatograph (UHPLC) coupled to a Sciex 4500 triple Quadrupole (Sciex, Mulgrave, Victoria, Australia) with an Electro Spray Ionisation (ESI) source operated in positive ionisation mode was used. Multiple Reaction Monitoring (MRM) was used for acquisition. An Agilent Poroshell 120 EC-C8 (2.1 × 100 mm, 2.7 μm, Agilent Technologies, Forest Hill, Australia) equipped with an inline filter was used to perform the chromatographic separation. The binary mobile phase consisted of 10 mM ammonium formate buffer, 5 % acetonitrile in Milli-Q water adjusted to pH 4 with formic acid (Solvent A) and 5 % v/v Milli-Q water (Solvent B). The column temperature was set at 50 °C, the column flow rate was 0.4 mL/min, and the temperature of the autosampler was kept at 4 °C throughout the entire analysis. The total run time of analysis was 15 min. The gradient was optimised at 10 %–20 % B in 0.50 min, held at 20 % B for 1 min, at 20 %–70 % in 6.50 min, at 90 % in 0.10 min and held for 1.90 min. The column was equilibrated for 5 min at 10 % B to decrease the possibility of carry-over and salt precipitation between injections.

The collision energy (CE) was optimised by flow injection of single standard solutions of every derivativatised sulfur compound at a concentration of 1 mg/L. The optimisation was performed to achieve the maximum product-ion intensity (MS/MS full scan). The flow injection allowed the selection of the optimal fragmentation voltage for precursor and product ion sensitivity. Two transitions per analyte were chosen and the pair that showed the highest sensitivity for each analyte was selected as the quantifier ion for the quantitation, whereas the other transition with lower sensitivity was selected as the qualifier ion. The ESI source parameters in the optimised method were the following: curtain gas (CUR) was set at 30 psi, collision gas (CAD) at 8 psi, Ion Spray Voltage (IS) at 5500 V, temperature at 600 °C, Ion Source Gas 1 (GS1) and Ion Source Gas 2 (GS2) were set at 60 °C and 70 °C, respectively. The ESI source parameters in the optimised method were the following: curtain gas (CUR) was set at 30 psi, collision gas (CAD) at 8 psi, Ion Spray Voltage (IS) at 5500 V, temperature at 600 °C, Ion Source Gas 1 (GS1) and Ion Source Gas 2 (GS2) were set at 60 °C and 70 °C, respectively. The Declustering Potential (DP), Entrance Potential (EP) and Collision Cell Exit Potential (CXP) were set at 60 V, 10 V and 13 V, respectively. Collision Energy (CE) was set at 55 V for H_2_S derivatives whereas for all the other compounds was set at 46 V. The MS/MS data was acquired in advanced scheduled Multiple Reaction Monitoring (MRM) mode using Analyst 1.7.1 and all data processing was performed with Sciex OS-MQ Quant Processing and Reporting software.

### 2.6 GC-SCD analysis

The purity of the AATP standard was assessed using static headspace gas-chromatography sulfur chemiluminescence detection (HS GC- SCD) as described by Siebert et al. [17] using a 280 Agilent 7890B GC, coupled to an Agilent 8355 SCD and equipped with a Gerstel MPS2 XL (Lasersan) autosampler.

### 2.7 Method optimisation

#### 2.7.1 Matrix interferences on sulfhydryl derivatisation

Standard stock solutions prepared in ethanol were compared to the standard stock solutions in an aqueous buffer matrix containing 12 % ethanol, 1 mM EDTA, 0.05 % formic acid and Milli-Q water. The ideal conditions to achieve complete derivatisation of sulfhydryls in wine matrices were achieved using the 12 % ethanol buffer matrix. Derivatising agents FEM and FMEA were added in molar excess to the sulfur constituents in the matrix to ensure complete derivatisation.

The pH of the solution during derivatisation was also investigated to maintain ideal conditions for derivatisation. The optimal pH for the derivatisation of sulfhydryl compounds was determined by adding 1 mM and 1 M ammonium bicarbonate solutions during derivatisation to 11-point calibration samples which included each analyte.

#### 2.7.2 Evaluation of blocking reagents to bind FEM and FMEA

Four blocking reagents, 1-pentanethiol, AATP, thiourea, and urea, were evaluated for their effectiveness to bind and remove excess FEM and FMEA without interfering with the derivatisation and analysis of the sulfhydryls quantified using this approach. The optimal blocking agent and relevant concentration of the blocking agent required were identified by comparing their interferences with the quantification of FEM and FMEA derivatives and ensuring the complete derivatisation of VSCs.

### 2.8 Method application

Commercial, bottled wines all sealed with screw cap closures of various vintages (2012–2020) and varieties (Syrah, Pinot Noir, Tempranillo, Merlot, Grenache, Mourvèdre, Chardonnay, Petit Chablis, Viognier, Sauvignon Blanc, Chardonnay, Rose) were sourced from Australian producers as well as one wine (Petit Chablis) from a French producer. The wines were analysed in 2023 and were selected at random and the wines were not preselected or screened for ‘reductive’ characters. The wines did not display any oxidised characters.

### 2.9 Statistical analysis

GraphPad 8 for Windows (GraphPad Software, San Diego, USA) was used for graphing and statistical analysis. Pearson correlation analysis was performed to investigate the correlation between MeSH and TCEP-releasable CH_3_–S-R as well as the correlation between EtSH and TCEP-releasable CH_3_–CH_2_–S-R. A two-tailed test was performed with a confidence interval of 95%. *P*-values are reported as * *P* < 0.05; **P < 0.01; ***P < 0.005.

## 3 Results and discussion

### 3.1 Method development and optimisation

The method originally described by Seiwert and Karst [30] for the analysis of large molecular and non-volatile sulfur compounds present in mg/L concentration ranges, namely cysteine, glutathione, cysteinyl glycine, *N*-acetylcysteine, homocysteine, and their disulfides in aqueous samples (urine, pH 7) was significantly adapted for the analysis of highly volatile and highly reactive VSC compounds that are present in wine at significantly lower concentrations (μg/L concentrations) compared to the concentrations of cysteine and cystine in urine (mg/L concentrations) and at low pH (generally pH 3 to pH 4). The autoxidation of the highly reactive sulfhydryls that may be facilitated by the presence of trace amounts of redox-active metals, such as copper and iron, was prevented by preparing all stock and working solutions of sulfhydryls and disulfides in a buffer solution containing EDTA. The risk of losing the highly volatile VSCs through volatilisation during the derivatisation process was decreased by preparing all stock and working solutions with cold (4 °C) Buffer A. All solutions were kept cold (4 °C) throughout the derivatisation steps. Wine samples were cooled down (4 °C) overnight before sampling.

The other major method development phase was ensuring that the derivatisation agents, FEM and FMEA, successfully and completely reacts with the sulfhydryls in wine matrices with pH ranging between pH 3 to 4. This was an important consideration considering that the matrix pH significantly affects the derivatisation reaction between maleimides and nucleophiles. The reaction between maleimides, such as FEM and FMEA, and sulfhydryls is a Michael addition of the thiolate anion to the double bond of the maleimide [31]. This reaction is pH sensitive, and for the chemoselective reaction of the maleimide with the thiols it is important to keep the pH of the matrix between 6.5 and 7.6 [32]. At pH 7.0 the reaction rate of maleimide with sulfhydryls is approximately 1000 times greater than the reaction rate with maleimides with amines [30,32]. However, at lower pH, the maleimides show no selectivity for their reaction with sulfhydryls compared to other functional groups such as amino groups and hydroxyl groups [30,32]. The method was thus adapted to include a basification step using an ammonium bicarbonate solution (1 M). This ensured the complete reaction between FEM and FMEA with all the sulfhydryls measured using this method (Table S1).

Additionally, it was found that AATP, the compound used as a blocking agent to remove excess FEM and FMEA in the original method described by Seiwert and Karst [30], contained large amounts of sulfur residues which generated H_2_S during the derivatisation process. Investigations into the purity of the AATP showed that the certification of analysis stated that the product contained up to 10 % impurities, and on close inspection, the AATP crystals had a pale yellow colour which also suggests impurities considering that AATP crystals should be pure white in colour. Solutions of AATP (3.0 g/L) were prepared in Buffer A and although AATP was only partially soluble in Buffer A, it was determined that a 3.0 g/L AATP solution generated 30 μg/L of H_2_S using the method described by Siebert et al. [17] (Figure S3). Considering that the concentration range of H_2_S in wine is 1–35 μg/L, AATP significantly interfered with the quantification of H_2_S and alternative FEM and FMEA binding agents were considered. Urea, thiourea and 1-pentanethiol were subsequently explored as alternative blocking agents to replace AATP, as both are highly soluble in both water and ethanol and it is known that they readily react with maleimides [33]. It was found that urea outperformed as a blocking agent when considering significant interferences of thiourea and 1-pentanethiol with the quantification of H_2_S and FMEA derivatives, respectively (Figure S4).

### 3.2 Differential labelling of sulfhydryls and disulfides

**Fig. 1:**
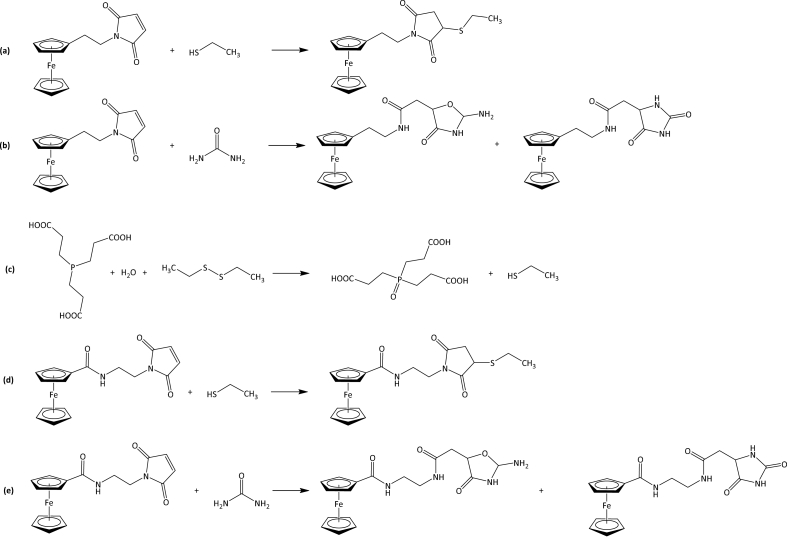
(a) Derivatisation of ethanethiol with FEM, (b) reaction of the FEM excess with urea, (c) reduction of diethyldisulfide with TCEP, (d) derivatisation of the produced ethanethiol with FMEA, (e) reaction of the excess FMEA with urea.

The simultaneous determination of free low molecular mass sulfhydryls and disulfide-bound sulfhydryl species present in the liquid phase of wine was achieved by the sequential labelling of free and bound sulfhydryl functionalities with two ferrocene-based maleimide reagents, namely FEM and FMEA (Fig. 1). Commercially available disulfides (DMDS and DEDS) were selected for this study to represent the pool of CH_3_–S-R and CH_3_–CH_2_–S-R TCEP-releasable sulfhydryls present in the wine. Samples were first treated with FEM to allow for the identification and quantification of the sulfhydryls present in the liquid phase of the wine (Fig. 1a). The excess FEM was then removed through the reaction with urea (Fig. 1b), which is a similar reaction to using AATP as a blocking agent to bind excess FEM [30]. The theoretical reaction products for the interaction between FEM and urea are shown in Fig. 1b as proposed by Achoui et al. [33]. The disulfides present in the matrix were then reduced with TCEP (Fig. 1c), followed by derivatisation with the second derivatisation agent, FMEA (Fig. 1d), and urea was again used to remove excess FMEA (Fig. 1e).

The derivatisation of individual stock solutions of VSCs was performed using FEM and FMEA as the derivatisation agent. During method validation solutions containing the sulfhydryl and disulfide standards evaluated in this study were first derivatised with FEM, and the reaction products were evaluated which proved the specificity of the derivatisation of only sulfhydryls and showed that the disulfides remained unaffected by FEM derivatisation. This information assisted in establishing the MRM transitions for each VSC, optimising sensitivity and chromatographic separation. The reaction products were assessed using HPLC-MS/MS to identify the derivatives and optimize the chromatographic separation.

### 3.3 MS/MS fragmentation of FEM and FMEA derivatives

**Fig. 2:**
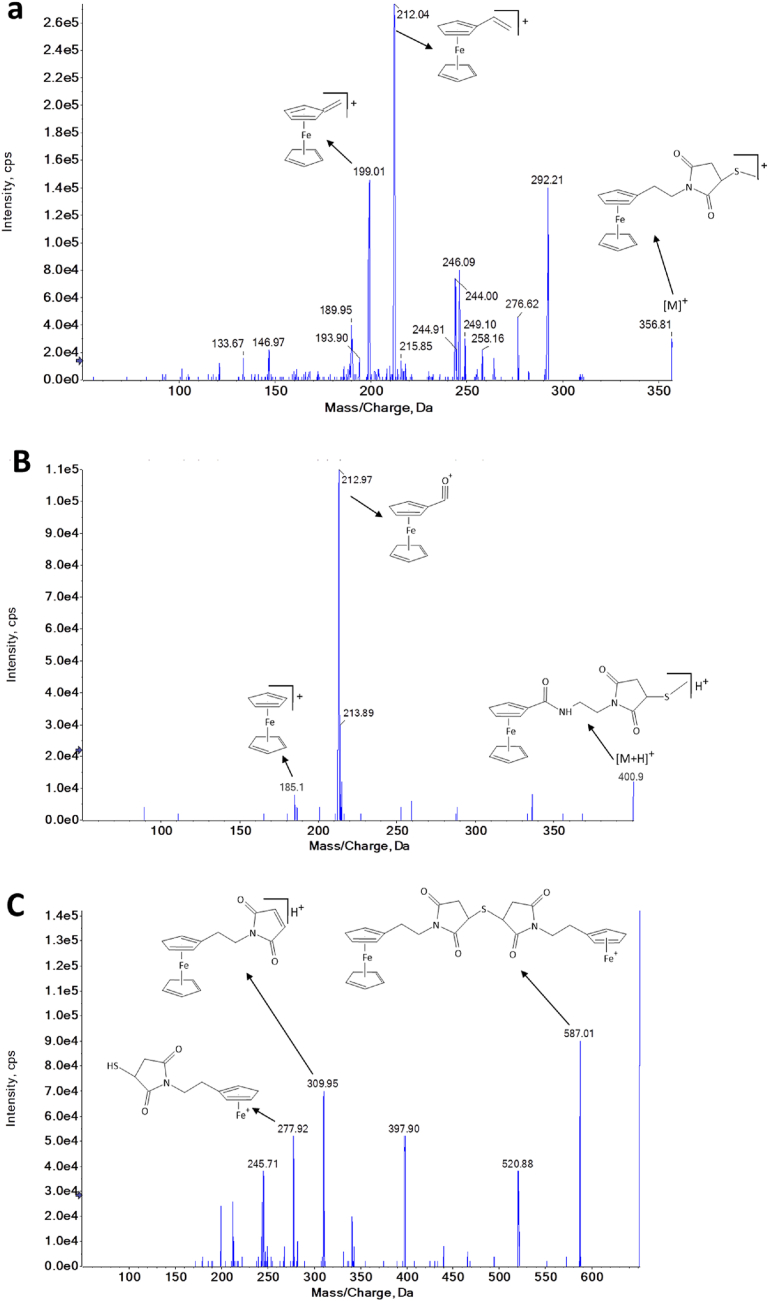
Product ion spectra of the (a) methanethiol derivative of FEM; (b) dimethyl disulfide detected as methanethiol FMEA derivative; and (c) hydrogen sulfide derivative of 2 × FEM.

For all analysed FEM and FMEA derivatives in this study, molecular ions [M]^+^ and [M + H]^+^ were detected respectively in the ESI mass spectrum. The fragment ion spectra of FEM derivatives resulted in two common product ions at *m*/*z* 199 and 212 (Fig. 2a). The ion at *m*/*z* 199 is also a product of the parent ion at *m*/*z* 356 that forms through the cleavage of the carbon-to-carbon bond adjacent to ferrocene. The greatest sensitivity for FEM derivatives was achieved by quantifying the derivatised products when *m*/*z* 212 was selected for the product ion. Thus, the product ion at *m*/*z* 199 was used for qualitative analysis and the product ion at *m*/*z* 212 was used for quantitative analysis. The FMEA derivatives provided the same product ions at *m*/*z* 213 and 185 (Fig. 2b). The major product ion at *m*/*z* 213 was used for quantitative analysis formed by the cleavage of the amide bond attached to the carbon atom of the carbonyl group. Further loss of carbon monoxide leads to product ion at *m*/*z* 185, which was used for qualitative analysis. Derivatised H_2_S yielded a different fragmentation pattern, because of its reaction with two FEM moieties, producing peaks at *m*/*z* 309.9, 587.0, and 277.9. A major fragment ion of derivatised H_2_S was present at *m*/*z* 309.9, which indicates the loss of the FEM group. A loss of 65 as a neutral loss is characteristic of the cyclopentadienyl ring loss from the parent ion to give 587.0. The loss of 309.9 indicates the loss of the FEM group is also observed from the ion at 587.0. The fragment pattern of derivatised H_2_S was calculated in Fig. 2c. After the elucidation of the fragmentation pattern, the mass parameters including the declustering potential and collision energy were optimised to obtain the maximum peak area for 14 sulfhydryl and disulfide derivatives including labelled references. The selected MS/MS parameters are given in the Materials and Method Section.

### 3.4 Method validation

**Fig. 3:**
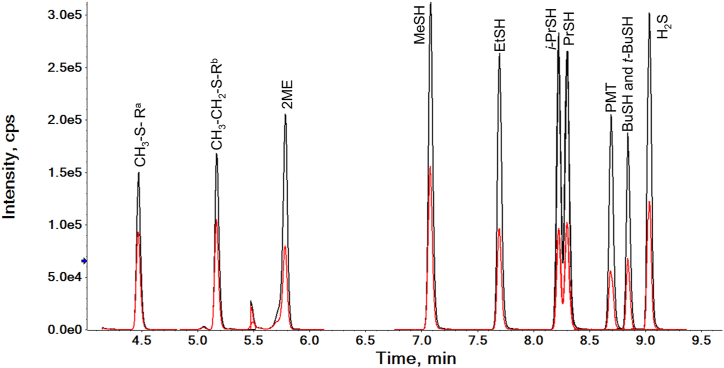
Chromatogram for the separation of a mixture of FEM and FMEA derivatives in a model wine solution containing CH_3_–S-R species, CH_3_–CH_2_–S-R species, 2-mercaptoethanol (2 ME), methanethiol (MeSH), ethanethiol (EtSH), *iso-*propanethiol (*i*-PrSH), propanethiol (PrSH), phenylmethanethiol (PMT), butylthiol (BuSH), *tert*-butylthiol (*t*-BuSH), and hydrogen sulfide (H_2_S), each at 25 μg/L, showing the quantification (black) and confirmation (red) MRM transitions. ^a & b^ as described in Table 1. (For interpretation of the references to colour in this figure legend, the reader is referred to the Web version of this article.)

The specificity of the HPLC−MS/MS method was evaluated by analysing a mixture of all the sulfhydryl and disulfide derivatives in a single chromatographic run. Efficient separation of target analytes was obtained within a retention window of 4.6 min with relative standard deviation of retention time never exceeding 0.4 %. The HPLC-MS/MS chromatogram corresponding to quantification and confirmation product ions of reference compounds spiked into a model wine solution is shown in Fig. 3. The specificity was further assured by measuring the ratio of the quantification compared to the confirmation product ion signals for each target analyte over model, white and red wine matrices. The coefficient variation of ion ratio on different days did not vary by more than 3.4 CV %, thus allowing the ion ratio of these transitions to be used as a confirmation of the identity of each analyte. Table 1 illustrates the MS transitions, their product ion ratios, and the retention times of each target analyte and the labelled internal standards (IS), and also shows which IS was used to quantify which analyte.

**Table 1:** Mass transitions, product ion ratios, and retention times for UHPLC-MS/MS analysis of derivatised sulfhydryls and TCEP-releasable CH_3_–S-R and CH_3_–CH_2_–S-R species using multiple reaction monitoring (MRM).

Analytes and internal standards	Mass transition (*m/z* → *m/z*)	RT^a^ ± SD^b^	Ion ratio (CV %)	Internal Standard used to quantify the analyte
CH_3_–S-R^a^	400.9 → 213.0	4.5 ± 0.01	0.68 (2.0%)	*d6*-DMDS
	400.9 → 185.1			
*d6*-DMDS	403.9 → 213.0	4.4 ± 0.01	0.64 (2.1%)	n/a
	403.9 → 184.9			
CH_3_–CH_2_–S-R^b^	415.0 → 213.0	5.2 ± 0.01	0.61 (2.0%)	*d6*-DMDS
	415.0 → 185.0			
2 ME	386.8 → 212.0	5.8 ± 0.01	0.39 (2.0%)	*d5*-EtSH
	386.8 → 199.0			
MeSH	356.8 → 212.0	7.1 ± 0.01	0.49 (1.8%)	*d5*-EtSH
	356.8 → 199.0			
EtSH	370.8 → 212.2	7.7 ± 0.01	0.37 (2.0%)	*d5*-EtSH
	370.8 → 199.3			
*d5*-EtSH	375.8 → 212.0	7.7 ± 0.01	0.41 (1.3%)	n/a
	375.8 → 199.0			
PrSH	384.9 → 212.1	8.3 ± 0.03	0.39 (2.7%)	*d5*-EtSH
	384.9 → 199.0			
*i*-PrSH	384.9 → 212.1	8.2 ± 0.03	0.3.5 (2.7%)	*d5*-EtSH
	384.9 → 199.0			
PMT	432.8 → 212.0	8.7 ± 0.03	3.4	*d5*-PMT
	432.8 → 199.1			
*d5*-PMT	437.8 → 212.0	8.7 ± 0.01	2.3	n/a
	437.8 → 199.1			
BuSH and *t*-BuSH	398.8 → 211.9	8.8 ± 0.03	3.2	*d5*-EtSH
	398.8 → 199.0			
H_2_S	651.9 → 309.9	9.0 ± 0.01	2.0	*d5*-EtSH
	651.9 → 587.0			

^a^RT, Retention time.

^b^SD, Standard deviation.

^a^Terminal methyl and R represents an organic substituent bound to a sulfur bridging atom.

^b^Terminal ethyl and R represent an organic substituent bound to a sulfur bridging atom.

The selectivity of the derivatisation agents was assessed to determine the ability of the method to distinguish the analytes of interest from other substances present in the matrices used to prepare the samples as well as in the various wine matrices. For this method this was achieved by comparing the chromatograms for derivatised blank model wine and blank model wine spiked with the target analytes of known concentrations (Figure S5) and no significant interferences were observed when quantifying the analytes of interest.

The analytical method was further validated within the typical dynamic range of target analytes in wine using model, red, and white wine matrices. The validation parameters included linearity, LOD, LOQ, recovery, and precision. Six-point calibration plots based on derivatised sulfhydryls and disulfide reference solutions at different concentrations were applied for the quantification of these analytes in wine matrices. The calibration plotted the ratio of the reference peak area to its corresponding labelled reference peak area compared to the spiked levels. Statistics describing the linearity are summarised in Table 2. As can be seen, determination coefficients (R^2^) higher than 0.99 were found for all target sulfhydryls and disulfides, indicating excellent linearity. Statistical analysis was performed on the slope and the intercept of the calibration curves gave satisfactory CV values below 17 % for all the target analytes with only one value at 24 % for the slope of the H_2_S calibration plot. The LOQ was defined as the lowest concentration with a coefficient variation of repeatability being ≤20 % and a percentage accuracy of 80–120 %, where a signal-to-noise (S/N) ratio above 10 was obtained for the corresponding peak. To determine the LOD, the noise was first measured over 10 injections of the blank model wine and the LOD was then calculated as the concentration for which the S/N ratio was greater than 3 [34]. The LOD and LOQ values ranged from 0.03 to 0.50 μg/L and 0.10–1.03 μg/L, respectively (Table 2). The LOQ values obtained are in good agreement with those reported for similar analytical methods. The LOQ determined for this method without sample preparation and preconcentration compares exceptionally well to the method described by Siebert et al. [17] where VSC LOQs range from 0.5 to 2.0 μg/L using a static headspace (HS) injection GC-SCD methodology. The methods described by Lopez et al. [21], Franco-Luesma et al. [35], and Ferreira et al. [11] do not specify LOQ values. However, for Franco-Luesma's solid phase microextracion (SPME) GC-PFPD method [7] the lowest reported MeSH value was 2.3 μg/L. The lowest standard curve points for H_2_S and MeSH described in the Ferreira et al. [11] method were 0.8 μg/L and 0.2 μg/L, respectively. Similarly, Nguyen et al. [16] reported LOQs for a range of VSCs of between 0.1 and 0.4 μg/L. The only compounds that have a higher LOQ than other reported methods, are PMT with a LOQ of 0.53 μg/L compared to the LOQ of 1.2 ng/L [24].

**Table 2:** LOD, LOQ, and linearity of calibration curves of 10 sulfhydryls and disulfide standards prepared in model wine by HPLC-MS/MS.

Analyte	Calibration range (μg/L)	Linear equation y = a (±SD) + b (±SD)	Linearity (R^2^)	LOD (μg/L)	LOQ (μg/L)	LOQ (μg/L) uncertainty (n = 4)
DMDS	0.53–212.00	y = 0.008 (±0.001) + 1.3e−02 (±2.2e-03)	0.999	0.11	0.53	0.03
DEDS	0.50–198.00	y = 0.015 (±0.003) + 2.1e−03 (±3.2e-04)	0.998	0.10	0.50	0.02
2 ME	0.56–222.80	y = 0.015 (±0.001) + 1.4e−04 (±2.1e−05)	0.998	0.11	0.56	0.02
MeSH	0.51–199.41	y = 0.026 (±0.003) + 1.7e−03 (±1.9e−04)	0.999	0.10	0.51	0.02
EtSH	0.54–215.43	y = 0.018 (±0.001) + 6.4e−04 (±2.7e−05)	0.999	0.11	0.54	0.02
*i*-PrSH	0.10–50.88	y = 0.028 (±0.002) + 1.5e−03 (±8.2e−05)	0.998	0.02	0.10	0.00
PrSH	0.11–52.50	y = 0.028 (±0.002) + 1.1e−03 (±9.7e−05)	0.998	0.04	0.11	0.00
PMT	0.53–105.80	y = 0.024 (±0.004) + 1.8e−03 (±5.8e−05)	0.998	0.1	0.53	0.01
BuSH and *t*-BuSH	0.10–209.25	y = 0.016 (±0.002) + 5.1e−04 (±7.5e−05)	0.998	0.04	0.10	0.01
H_2_S	1.03–205.61	y = 0.025 (±0.006) + 2.4e−03 (±3.3e−04)	0.999	0.5	1.03	0.03

^a & b^ as described in Table 1.

The repeatability was calculated using the signal of labelled reference sulfhydryl and disulfide spiked into model, red, and white wine samples before derivatisation. The coefficient of variation values are depicted in Table S2 of the Supporting Information. Values of CV % were always lower than 9.8 % for all the wine matrices. The intraday and interday precision and accuracy were also fully evaluated by analysing white and red wine samples on different days by different analysts, spiked with sulfhydryls and disulfides at two concentration levels of 5 and 50 μg/mL. For intraday evaluations, the samples were analysed seven times in one day, and for the interday evaluations, the samples were analysed once per day on six different days over a two-week period. Table 3 summarises the precision and accuracy statistics of the method for all the target analytes. The average coefficient variation of intraday precision for the majority of target analytes at both low and high levels was approximately 4.38 CV % in real wine samples, and none greater than 18.6 %. For the majority of analytes, the interday precision at each level was ≤14.9 CV % for both white and red wine samples, however, the intraday precision for H_2_S was lowest at high concentrations in both red (15.9 % CV) and white wine (15.8% CV) and reached up to 37.8 % CV at low concentrations in red wine. It has previously been reported that wine matrix compounds scalp H_2_S when it is added to wine [21] which affects the quantification of added H_2_S, but not the quantification of H_2_S naturally present in wine. Regarding accuracy, the experimental result was typically within 80–125 % of the exact value for all the derivatives except for H_2_S, which presented slightly lower recoveries at low levels in white wine samples. In the case of red wine samples, a moderate decrease in H_2_S recovery was observed at both low and high spiked levels. The poor recovery of H_2_S spikes in real wine samples was obtained possibly due to the scalping of H_2_S by metals or potentially electrophiles such as oxidised phenolics that are naturally present in the wine matrix [20]. The stability of derivatives in model wine solution was also tested. There was no significant decrease in the peak area of target analytes after storing at – 20 °C for 20 days, suggesting acceptable storage stability.

**Table 3:** Intra- and inter-day precision and accuracy for determination of 10 sulfhydryls and TCEP-releasable CH_3_–S-R and CH_3_–CH_2_–S-R species in white and red wine matrices by developed HPLC-MS/MS.

Analyte	Spiked amount (μg/L)	White Wine Matrix				Red Wine Matrix			
		Intra-day (n = 7)		Inter-day (n = 6)		Intra-day (n = 7)		Inter-day (n = 6)	
		CV (%)	Recovery (%)	CV (%)	Recovery (%)	CV (%)	Recovery (%)	CV (%)	Recovery (%)
CH_3_–S- R^a^	5	8.9	97.6	14.9	94.3	17.0	104.2	8.8	98.9
	50	2.3	96.7	8.9	94.5	2.5	96.3	7.2	91.8
CH_3_–CH_2_–S–R^b^	5	2.5	81.5	7.3	87.1	3.1	91.8	6.0	92.2
	50	2.0	80.8	4.4	83.2	1.7	95.3	5.6	89.5
2 ME	5	18.6	93.0	12.1	100.9	16.3	124.7	13.3	112.8
	50	8.6	98.2	13.8	102.2	2.6	116.8	12.8	104.9
MeSH	5	6.7	92.2	11.8	97.7	13.6	112.2	10.4	104.6
	50	2.2	92.4	11.7	92.9	1.2	105.5	6.0	101.5
EtSH	5	3.0	90.7	5.7	97.9	2.2	97.6	6.4	103.0
	50	1.2	90.4	12.2	94.0	1.0	99.9	10.0	102.1
PrSH	5	2.8	118.0	5.4	113.7	2.0	101.9	6.3	100.5
	50	1.9	106.6	6.2	109.9	1.1	91.3	7.4	98.5
*i*-PrSH	5	2.8	118.0	6.7	113.0	2.0	101.9	10.8	92.5
	50	1.9	106.6	10.9	107.9	1.1	91.3	6.8	94.0
PMT	5	2.1	123.8	3.3	118.0	2.9	110.7	4.9	107.7
	50	2.2	116.6	5.7	113.6	2.0	107.2	6.1	106.7
BuSH and *t*-BuSH	5	2.6	118.2	4.8	112.8	2.7	95.2	5.6	92.7
	50	1.9	125.0	4.0	116.5	1.8	102.4	13.5	99.1
H_2_S	5	4.1	67.9	28.5	72.7	6.9	49.7	37.8	49.3
	50	2.4	100	22.8	85	4.0	57.9	15.9	59.0

^a&b^As described in Table 1.

In summary, experiments demonstrated that the new method is selective, linear, precise, accurate, and stable, and can be used for the simultaneous determination of sulfhydryls and disulfides in red and white wine samples.

### 3.5 Quantitative analysis of volatile sulfur compounds in a range of wine

The optimised method was used to determine the concentration of eight sulfhydryls and two disulfide moieties in 14 commercial red and white wines. The wines were not selected or screened for ‘reduced’ aromas and were selected at random for this study. Wines were cooled to 4 °C overnight before analysis. Table 4 shows the concentration ranges of compounds determined for each variety that was studied.

**Table 4:** Calculated concentration of target sulfhydryls and TCEP-releasable CH_3_–S-R and CH_3_–CH_2_–S-R species in real wine samples.

Sample	MeSH	EtSH	CH_3_–S-R	CH_3_–CH_2_–S-R	PMT	2 ME	*i*-PrSH	PrSH	BuSH	H_2_S
RW1	12.7	<LOQ	88.1	<LOQ	N.D.^a^	16.4	N.D.	N.D.	N.D.	13.3
RW2	5.8	0.8	51.5	4.3	N.D.	17.7	N.D.	N.D.	N.D.	10.3
RW3	10.1	<LOQ	22.7	<LOQ	N.D.	72.3	N.D.	N.D.	N.D.	64.6
RW4	5.9	<LOQ	53.7	<LOQ	N.D.	27.9	N.D.	N.D.	N.D.	4.5
RW5	7.2	<LOQ	32.2	<LOQ	N.D.	30.8	N.D.	N.D.	N.D.	9.6
RW6	15.6	<LOQ	80.9	<LOQ	N.D.	13.2	N.D.	N.D.	N.D.	6.2
RW7	6.4	<LOQ	33.3	<LOQ	N.D.	31.5	N.D.	N.D.	N.D.	9.4
WW1	6.6	1.5	25.5	4.6	N.D.	86.3	N.D.	N.D.	N.D.	10.5
WW2	6.6	1.7	17.1	2.7	N.D.	108.3	N.D.	N.D.	N.D.	10.7
WW3	2.5	0.5	5.2	0.5	N.D.	84.3	N.D.	N.D.	N.D.	6.4
WW4	5.2	<LOQ	10.7	<LOQ	N.D.	64	N.D.	N.D.	N.D.	7.8
WW5	14	2.4	19.7	2.1	N.D.	72.5	N.D.	N.D.	N.D.	8.3
WW6	4.2	<LOQ	20.6	<LOQ	N.D.	6.4	N.D.	N.D.	N.D.	8.7
WW7	11	<LOQ	32.4	0.9	N.D.	65.4	N.D.	N.D.	N.D.	26.4

a Not detected.

**Fig. 4:**
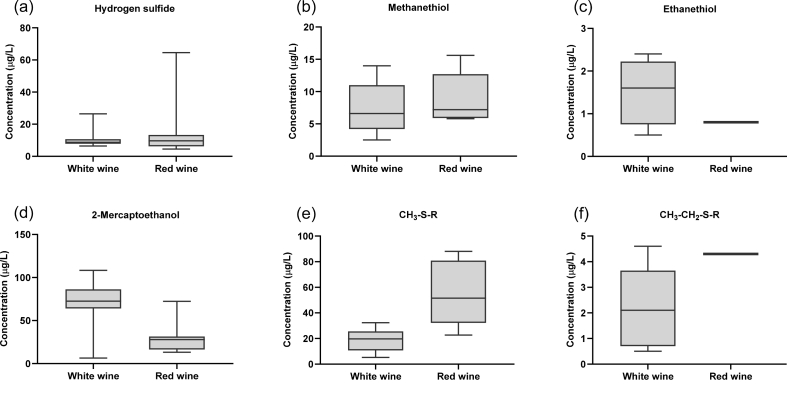
Concentrations (μg/L) of (a) hydrogen sulfide, (b) methanethiol, (c) ethanethiol, (d) 2-mercaptoethanol, (e) CH_3_–S-R species and (f) CH_3_–CH_2_–S-R species measured in seven commercial white wines and seven commercial red wines.

Hydrogen sulfide was detected in all the wines (Fig. 4). The aroma descriptors for H_2_S are ‘rotten egg’, ‘sewage-like’, and ‘vegetal’ [17]. The lowest and the highest levels of H_2_S in white wine were 6.4 μg/L and 26.4 μg/L, respectively, as measured in two different bottles of Chardonnay wine sourced from two different producers from different winemaking regions (Table 4). Similarly, the lowest and the highest levels of H_2_S in red wine were 4.5 μg/L and 64.6 μg/L, respectively, as measured in two different bottles of Shiraz wine sourced from two different producers from different winemaking regions (Table 4). This demonstrates that the presence of H_2_S in wine is not related to variety alone, but it is significantly affected by winemaking styles, winemaking additives, and winemaking conditions. The average concentration of H_2_S in white wine and red wine was 11.3 μg/L and 16.8 μg/L, respectively. These values detected in the liquid phase of the 14 wines analysed using the derivatisation method described in this study, are comparable to H_2_S concentrations measured in the headspace of wines using a salt treatment method [17]. This is a strong indication that this particular method not only quantifies “free” sulfhydryls, but also quantifies the metal-bound sulfhydryls. When H_2_S is present at concentrations higher than its aroma threshold (1.1 and 1.6 μg/L in red and white wine [17]) and when H_2_S is unmasked by other wine aromatic compounds, would its presence correspond to the observation of undesirable aroma traits, such as ‘rotten egg’ or ‘sewage-like’ aromas. Recent studies have demonstrated that the perception of the negative aroma imparted by H_2_S is strongly correlated to the presence of copper in wine and that the speciation of copper is a significant indicator of whether H_2_S would be present in ‘free’ or ‘metal-bound’ forms [7]. Only the “free” forms of H_2_S contribute to the perception of ‘reductive’ faults [7]. The ability of the current LC-MS/MS method to differentiate between “free” and “metal-bound” forms of H_2_S is yet to be established, however, the fact that the H_2_S concentrations measured using this method are comparable to those measured in wine using a brine/salt addition, is a strong indicator that the sulfhydryls bound to metals are also quantified in the sulfhydryl fraction.

The aroma of MeSH can be described as ‘rotten cabbage’, ‘burnt rubber’, and ‘putrefaction’. Arguably, MeSH has a greater contribution to ‘reductive’ aromas in wine than H_2_S. Interestingly, the average concentrations of MeSH were similar in the red wines and white wines analysed using this method, at 9.1 μg/L and 7.2 μg/L, respectively (Table 4, Fig. 4). A wide range of MeSH concentrations were measured in the different wine samples, with the minimum values of MeSH were 5.8 μg/L and 2.5 μg/L in red and white wine, respectively, and the maximum values of MeSH were 15.6 μg/L and 14.0 μg/L in red and white wine, respectively. This is in agreement with results obtained by previous studies that analysed the headspace of wine using gas chromatographic techniques [17,19]. As was the case with H_2_S concentrations, the MeSH measured using this method is comparable to those measured in wine using a brine/salt addition, which is a strong indicator that sulfhydryls bound to metals are also quantified in the “ulfhydryl fraction [7,36]. This compound was present in all wines analysed in this study, which is also in agreement with previous studies. Considering that the aroma detection threshold for MeSH is low (1.8–3.1 μg/L, [17]) this demonstrates that all wines that contain MeSH would not necessarily present with ‘reductive’ off-aromas and that the perception of MeSH is highly dependent on the matrix of the wine.

Four white wines contained EtSH and only one red wine contained EtSH (Table 4, Fig. 4). The aroma perception of EtSH can be described as ‘onion’, ‘rubbery’, ‘burnt match’, ‘sulfidy’, and ‘earthy’ [17]. The average concentration of EtSH was considerably low at approximately 1 μg/L for both white and red wine, which is the aroma threshold of EtSH in wine. Previous studies have shown that EtSH is rarely detected in the headspace of wine [17,19], however, when EtSH is present in wine above its aroma threshold of 1.1 μg/L [17] the wines always present with ‘reductive’ off-aromas [37].

2-Mercaptoethanol is classified as a “heavy VSC” and it imparts aromas of ‘solvent’, ‘barnyard’, and ‘burnt rubber’ [38]. It is usually present at concentrations lower than its aroma threshold of approximately 450 μg/L in white wine and 600 μg/L in red wine [39] and it is produced as a breakdown product of sulfur-containing amino acids. Even wine that is rated as ‘clean’ without any ‘reductive’ aromas usually contains 2 ME at concentrations of approx. 72 μg/L [38]. In accordance with the previously published data, in this study the average concentration for 2 ME was 69.6 μg/L in white wine and 30.0 μg/L in red wine. A maximum of 108 μg/L and 72.3 μg/L of 2 ME were measured in white and red wine, respectively (Table 4, Fig. 4).

A considerably large concentration of TCEP-releasable CH_3_–S-R and CH_3_–CH_2_–S-R species were identified in the wines screened using the method described in this paper. The average concentration for TCEP-releasable CH_3_–S-R was 18.7 μg/L in white wine and 51.8 μg/L in red wine (Table 4, Fig. 4). A maximum of 32.4 μg/L and 88.1 μg/L of TCEP-releasable CH_3_–S-R was measured in white and red wine, respectively (Table 4, Fig. 4). The average concentration for TCEP-releasable CH_3_–CH_2_–S-R was considerably lower than that of TCEP-releasable CH_3_–S-R, with an average TCEP-releasable CH_3_–CH_2_–S-R concentration of 2.2 μg/L in white wine and 4.3 μg/L in red wine (Table 4, Fig. 4). A maximum of approximately 4.0 μg/L were measured in both white and red wine samples (Table 4, Fig. 4). This method cannot differentiate between symmetrical disulfides or polysulfides such as CH_3_–S–S–CH_3_ or CH_3_–S_n_–CH_3_; and asymmetrical disulfides or polysulfides such as CH_3_–S–S–CH_2_–CH_3_ or CH_3_–S_n_–CH_2_–CH_3_ for example, however, by determining the concentration of TCEP-releasable sulfhydryls present in the liquid phase of wine, it may be possible to gauge a wine's ‘reductive’ potential by estimating the pool of putative precursors to VSCs associated with ‘reductive’ faults. Recent studies have shown that polysulfides can readily degrade and liberate aroma-active sulfur compounds such as H_2_S [26,40].

**Fig. 5:**
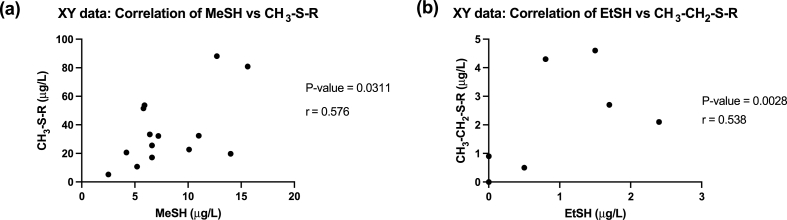
Pearson correlation between the concentrations (μg/L) of (a) methanethiol (MeSH) and TCEP-releasable CH_3_–S-R species, and (b) ethanethiol (EtSH) and TCEP-releasable CH_3_–CH_2_–S-R species measured in seven commercial white wines and seven commercial red wines.

The correlation between MeSH and TCEP-releasable CH_3_–S-R, as well as EtSH and TCEP-releasable CH_3_–CH_2_–S-R was evaluated using Pearson correlation analysis (Fig. 5). Pearson correlation analysis revealed a significant positive correlation between MeSH and TCEP-releasable CH_3_–S-R species (r = 0.576, *P*-value = 0.0311, Fig. 5a) as well as a significant positive correlation between EtSH and TCEP-releasable CH_3_–CH_2_–S-R species (r = 0.538, *P*-value = 0.0028, Fig. 5b). It is important to note that the number of wines used in this study is small (n = 14), and EtSH and CH_3_–CH_2_–S-R were only measured in six wines. However, in TCEP-releasable CH_3_–CH_2_–S-R species were only measured in wines containing EtSH in five out of six wines. This supports the correlation between these species even though the sample size of this study was small.

The correlation between wine age and TCEP-releasable species was also evaluated, however, no correlation was found between wine age and TCEP-releasable CH_3_–S-R species (r = −0.4861, *P*-value 0.125, Figure S6a) and TCEP-releasable CH_3_–CH_2_–S-R species (r = 0.1838, *P*-value 0.578, Figure S6b). However, the number of the wines analysed in this study was small and evaluating this correlation in a larger set of wines would provide valuable insight into the origin of the TCEP-releasable species and whether they originate from disulfides or polysulfides which concentrations should decrease over time due to sulfitolysis, unless the wines have been depleted from sulfites or were oxidised.

The methods described by Kreitman et al. [26] and Ferreira et al. [11] that quantified the total pool of H_2_S and MeSH present in wine used a similar reduction step utilising TCEP as was used in the current study. Only a small selection of wines was analysed in this current study (n = 14), in the Kreitman et al. [26] study (n = 6), and in the Ferreira et al. [11] study (n = 7), so the methods cannot easily be compared. Noting that all three studies involved different sets of wine samples, the concentration range of H_2_S quantified by Kreitman et al. [26] (51.9–79.6 μg/L) and Ferreira et al. [11] (42–154 μg/L, up to 7-day release) were considerably larger than the concentration range of H_2_S quantified in this study (4.5–64.6 μg/L). This might suggest that the new LC-MS/MS method described here may be further improved by implementing either Kreitman or Ferreira's conditions of either using copper (I) ligands to prevent copper from interfering with H_2_S quantification [26], or utilising the smart trapping of sulfhydryls with copper (I) and then releasing H_2_S in a secondary step [11]. Interestingly, MeSH concentrations quantified using these two methods are more comparable, which further supports the importance of copper (I) for the quantification of free H_2_S as well as the H_2_S liberated from its precursors.

## 4 Conclusions

A novel sulfhydryl and TCEP-releasable sulfhydryl derivatisation and quantification method has been developed, validated, and applied to a range of wine samples. Prior to LC-MS/MS analysis, free sulfhydryls were derivatised with FEM, and TCEP-releasable sulfhydryls were differentially labelling using FMEA as derivatisation agent. All sulfhydryls were derivatised in the wine samples after pH adjustment, samples were then directly injected without additional concentration steps, and analysed by HPLC-MS/MS in MRM mode.

One of the most important outcomes of the work is that this methodology can provide insights into the sulfhydryl and TCEP-releasable sulfhydryl species present in a wine. The data can provide information about a wine's risk of developing ‘reductive’ faults post-bottling and potentially during the ageing process. Significant positive correlations were found between MeSH and CH_3_–S-R TCEP-releasable species, and significant positive correlations were found between EtSH and CH_3_–CH_2_–S-R TCEP-releasable species.

To demonstrate the occurrence of the VSCs in wine, an analysis of a range of commercial white and red wines was conducted which revealed the presence of H_2_S, MeSH, EtSH, and 2 ME at aroma active concentrations. Furthermore, the quantification of the pool of TCEP-releasable CH_3_–S-R and CH_3_–CH_2_–S-R species provided important information on the pool of putative precursors to important VSC compounds associated with ‘reductive’ faults in wine.

This analytical method may find application in other food and beverage matrices, particularly where sulfhydryls play important roles in modulating the aroma and flavour of the products.

## Data availability

Data will be made available on request.

## CRediT authorship contribution statement

**Marlize Z. Bekker:** Writing – review & editing, Writing – original draft, Visualization, Validation, Supervision, Software, Project administration, Methodology, Investigation, Formal analysis, Data curation, Conceptualization. **Maryam Taraji:** Writing – review & editing, Writing – original draft, Visualization, Validation, Investigation, Formal analysis. **Vilma Hysenaj:** Writing – review & editing, Visualization, Validation, Methodology, Investigation, Formal analysis, Data curation. **Natoiya Lloyd:** Writing – review & editing, Writing – original draft, Project administration, Methodology, Formal analysis, Conceptualization.

## Declaration of competing interest

Dr Marlize Bekker reports financial support was provided by 10.13039/501100007915Wine Australia. If there are other authors, they declare that they have no known competing financial interests or personal relationships that could have appeared to influence the work reported in this paper.

## References

[1] Müller N., Rauhut D., Tarasov A. (2022). Sulfane sulfur compounds as source of reappearance of reductive off-odors in wine. Fermentation.

[2] Smith M.E., Bekker M.Z., Smith P.A., Wilkes E.N. (2015). Sources of volatile sulfur compounds in wine. Aust. J. Grape Wine Res..

[3] Goode J., Harrop S. (2008). Proceedings of the Les XXes Entretiens Scientifiques Lallamand.

[4] Swiegers J.H., Pretorius I.S. (2007). Modulation of volatile sulfur compounds by wine yeast. Appl. Microbiol. Biotechnol..

[5] Viviers M.Z., Smith M.E., Wilkes E., Smith P. (2013). Effects of five metals on the evolution of hydrogen sulfide, methanethiol, and dimethyl sulfide during anaerobic storage of Chardonnay and Shiraz wines. J. Agric. Food Chem..

[6] Ferreira V., Franco-Luesma E., Vela E., Lopez R., Hernandez-Orte P. (2018). Elusive chemistry of hydrogen sulfide and mercaptans in wine. J. Agric. Food Chem..

[7] Franco-Luesma E., Ferreira V. (2014). Quantitative analysis of free and bonded forms of volatile sulfur compounds in wine. Basic methodologies and evidences showing the existence of reversible cation-complexed forms. J. Chromatogr. A.

[8] Nikolantonaki M., Waterhouse A.L. (2012). A method to quantify quinone reaction rates with wine relevant nucleophiles: a key to the understanding of oxidative loss of varietal thiols. J. Agric. Food Chem..

[9] Kreitman G.Y., Elias R.J., Jeffery D.W., Sacks G.L. (2019). Loss and formation of malodorous volatile sulfhydryl compounds during wine storage. Crit. Rev. Food Sci. Nutr..

[10] Bekker M.Z., Wilkes E.N., Smith P.A. (2018). Evaluation of putative precursors of key 'reductive' compounds in wines post-bottling. Food Chem..

[11] Ferreira V., Sanchez-Gimeno D., Ontanon I. (2023). A method for the quantitative and reversible trapping of sulfidic gases from headspaces and its application to the study of wine reductive off-odors. Food Chem..

[12] Müller N., Rauhut D. (2018). Recent developments on the origin and nature of reductive sulfurous off-odours in wine. Fermentation.

[13] Tominaga T., Guimbertau G., Dubourdieu D. (2003). Contribution of benzenemethanethiol to smoky aroma of certain Vitis vinifera L. Wines. J. Agric. Food Chem..

[14] Malfeito-Ferreira M. (2022). Wine minerality and funkiness: blending the two tales of the same story. Fermentation.

[15] Espinase Nandorfy D. (2023). The role of potent thiols in “empyreumatic” flint/struck-match/mineral odours in Chardonnay wine. Aust. J. Grape Wine Res..

[16] Nguyen D.D., Nicolau P., Kilmartin P.A., Salih B. (2012). Gas Chromatography in Plant Science, Wine Technology, Toxicology and Some Specific Applications.

[17] Siebert T.E., Solomon M.R., Pollnitz A.P., Jeffery D.W. (2010). Selective determination of volatile sulfur compounds in wine by gas chromatography with sulfur chemiluminescence detection. J. Agric. Food Chem..

[18] Tan B. (2017). New method for quantification of gasotransmitter hydrogen sulfide in biological matrices by LC-MS/MS. Sci. Rep..

[19] Rauhut D., Kürbel H., MacNamara K., Grossmann M. (1998). Headspace GC-SCD monitoring of low volatile sulfur compounds during fermentation and in wine. Analusis.

[20] Ontanon I., Vela E., Hernandez-Orte P., Ferreira V. (2019). Gas chromatographic-sulfur chemiluminescent detector procedures for the simultaneous determination of free forms of volatile sulfur compounds including sulfur dioxide and for the determination of their metal-complexed forms. J. Chromatogr. A.

[21] Lopez R., Lapena A.C., Cacho J., Ferreira V. (2007). Quantitative determination of wine highly volatile sulfur compounds by using automated headspace solid-phase microextraction and gas chromatography-pulsed flame photometric detection. Critical study and optimization of a new procedure. J. Chromatogr. A.

[22] Starkenmann C., Chappuis C.J., Niclass Y., Deneulin P. (2016). Identification of hydrogen disulfanes and hydrogen trisulfanes in H(2)S bottle, in flint, and in dry mineral white wine. J. Agric. Food Chem..

[23] Frerot E., Bagnoud A., Cicchetti E. (2014). Quantification of hydrogen sulfide and methanethiol and the study of their scavenging by biocides of the isothiazolone family. Chempluschem.

[24] Capone D.L., Ristic R., Pardon K.H., Jeffery D.W. (2015). Simple quantitative determination of potent thiols at ultratrace levels in wine by derivatization and high-performance liquid chromatography-tandem mass spectrometry (HPLC-MS/MS) analysis. Anal. Chem..

[25] Jastrzembski J.A., Allison R.B., Friedberg E., Sacks G.L. (2017). Role of elemental sulfur in forming latent precursors of H(2)S in wine. J. Agric. Food Chem..

[26] Kreitman G.Y., Danilewicz J.C., Jeffery D.W., Elias R.J. (2017). Copper(II)-Mediated hydrogen sulfide and thiol oxidation to disulfides and organic polysulfanes and their reductive cleavage in wine: mechanistic elucidation and potential applications. J. Agric. Food Chem..

[27] van Leeuwen K.A., Nardin T., Barker D., Fedrizzi B., Nicolini G., Larcher R. (2020). A novel LC-HRMS method reveals cysteinyl and glutathionyl polysulfides in wine. Talanta.

[28] Chen Y., Jastrzembski J.A., Sacks G.L. (2017). Copper-Complexed hydrogen sulfide in wine: measurement by gas detection tubes and comparison of release approaches. Am. J. Enol. Vitic..

[29] Seiwert B., Karst U. (2007). Analysis of cysteine-containing proteins using precolumn derivatization with N-(2-ferroceneethyl)maleimide and liquid chromatography/electrochemistry/mass spectrometry. Anal. Bioanal. Chem..

[30] Seiwert B., Karst U. (2007). Simultaneous LC/MS/MS determination of thiols and disulfides in urine samples based on differential labeling with ferrocene-based maleimides. Anal. Chem..

[31] Gennari A., Wedgwood J., Lallana E., Francini N., Tirelli N. (2020). Thiol-based michael-type addition. A systematic evaluation of its controlling factors. Tetrahedron.

[32] Hermanson G.T. (2013).

[33] Achoui N., Zaioua K., Hammoutene D., Kolli-Nedjar B., Akacem Y. (2019). Interaction of thiourea and urea with maleimide: comparative theoretical DFT study. Heliyon.

[34] Sharma S., Goyal S., Chauhan K. (2018). A review on analytical method development and validation. Int. J. Appl. Pharm..

[35] Ferreira V., Bueno M., Franco-Luesma E., Cullere L., Fernandez-Zurbano P. (2014). Key changes in wine aroma active compounds during bottle storage of Spanish red wines under different oxygen levels. J. Agric. Food Chem..

[36] Franco-Luesma E., Ferreira V. (2016). Formation and release of H2S, methanethiol, and dimethylsulfide during the anoxic storage of wines at room temperature. J. Agric. Food Chem..

[37] Bekker M.Z., Day M.P., Holt H., Wilkes E., Smith P.A. (2016). Effect of oxygen exposure during fermentation on volatile sulfur compounds in Shiraz wine and a comparison of strategies for remediation of reductive character. Aust. J. Grape Wine Res..

[38] Ribéreau-Gayon P., Glories Y., Maujean A., Dubourdieu D. (2006).

[39] Lavigne V., Dubourdieu D. (1996). Demonstration and interpretation of the yeast lees ability to absorb certain volatile thiols contained in wine. J. Int. Sci. Vigne Vin.

[40] Bekker M.Z., Kreitman G.Y., Jeffery D.W., Danilewicz J.C. (2018). Liberation of hydrogen sulfide from dicysteinyl polysulfanes in model wine. J. Agric. Food Chem..

