# Supplementary material for: Accurate measurement of sulfhydryls and TCEP-releasable sulfhydryls in the liquid phase of wine that contribute to ‘reductive’ aromas using LC-MS/MS
Source: Heliyon. 2024 Apr 1;10(7):e28929. doi: 10.1016/j.heliyon.2024.e28929 (PMC11004803; doi:10.1016/j.heliyon.2024.e28929)
Supplement: Multimedia component 1 [file mmc1.pdf]

Supplementary Material for

Accurate measurement of sulfhydryls and TCEP-releasable sulfhydryls present in the liquid phase of wine that contribute to 'reductive' aromas using LC-MS/MS

Marlize Z. Bekker[a,b*], Maryam Taraji[a,c], Vilma Hysenaj[a,c], and Natoiya Lloyd[a,c]

[a] The Australian Wine Research Institute, P.O. Box 197, Glen Osmond, SA, 5064, Australia

[b] School of Agriculture and Food Sustainability, The University of Queensland, St Lucia, QLD 4067, Australia

[c] Metabolomics Australia, PO Box 197, Adelaide, SA 5064, Australia

*Corresponding author: Dr Marlize Bekker, telephone +61 07 3365 2123,

m.bekker@uq.edu.au

Table of Contents

	Page
**S1.1** Synthesis of N-(2-Ferroceneethyl) maleimide (FEM).	3
**Figure S1.** Synthesis of N-(2-ferroceneethyl) maleimide (FEM).	4
**S1.2** Synthesis of Ferrocenoylethylmaleimide (FMEA).	4
**Figure S2.** Synthesis of ferrocenecarboxylic acid-(2-maleimidoyl)ethylamide (FMEA).	5
**Figure S3.** Liberation of hydrogen sulfide (H_2S) from 4-acetaminothiophenol (AATP) standard (3.0 g/L) prepared in Buffer A.	6
**Figure S4.** Comparison of interferences of four blocking reagents including AATP, thiourea, 1-pentanethiol and urea with the quantitation of target sulfhudryls in model wine solution.	7
**Figure S5.** Selectivity in model wine solution. Chromatogram of derivatised blank model wine solution (red) and chromatogram of derivatised blank model wine containing CH_3-S-R species, CH_3-CH_2-S-R species, 2-mercaptoethanol (2ME), methanethiol (MeSH), ethanethiol (EtSH), *iso*-propanethiol (*i*-PrSH), propanethiol (PrSH), phenylmethanethiol (PMT), butylthiol (BuSH), *tert*-butylthiol (*t*-BuSH), and hydrogen sulfide (H_2S) at their LOQ levels (black).[a] is terminal methyl and R represents an organic substituent with a sulfur bridging atom and [b] is terminal ethyl and R represents an organic substituent with a sulfur bridging atom.	8
**Figure S6.** Pearson correlation between wine age and the concentrations (μg/L) of (a) TCEP-releasable CH_3-S-R species, and (b) TCEP-releasable CH_3-CH_2-S-R species measured in seven commercial white wines and seven commercial red wines.	8
**Table S1.** Comparison of peak area response for sulfhydryls and TCEP-releasable CH_3-S-R[a] and CH_3-CH_2-S-R[a] species in model wine by HPLC-MS/MS after derivatisation with FEM and FMEA.	9
**Table S2.** Intra- and inter-day repeatability for the analysis of sulfhydryls and TCEP-releasable CH_3-S-R[a] species in white and red wine matrices by HPLC-MS/MS after derivatisation with FEM and FMEA.	9

S1.1 Synthesis of N-(2-Ferroceneethyl) maleimide (FEM)

2-Ferroceneethyl amine. 2-Ferrocene acetonitrile, (2.563 g, 11.39 mmoles, 1eq) was dissolved in dry THF (25.3 ml) and this solution was added dropwise to a chilled and stirring suspension of anhydrous aluminium chloride (3.037 g, 22.777 mmoles, 2eq) and lithium aluminium hydride (1.249 g, 32.90 mmoles, 2.89eq) in dry THF (50 mL). After 2 hours at reflux under a nitrogen atmosphere the reaction mixture was chilled before being quenched with water (50 mL) added dropwise, followed by sodium hydroxide (10 M, 5 mL) The aqueous layer was extracted with diethyl ether (3 x 100mL) and the combined organic layers were washed water (2 x 10 mL) then brine (3 x 15 mL) before being dried with anhydrous sodium sulfate. Removal of the solvent en vacuo gave 1.710 g (66 % crude yield) of an orange solid. *^{1}H NMR ($CDCl_3$, δ ppm):* 4.11 (5H, s, Ferrocenyl); 4.09 (2H, s, Ferrocenyl); 4.07 (2H, s, Ferrocenyl); 2.84 (2H, s, H_1); 2.52 (2H, s, H_2); 2.21 (2H, br s, NH_2).

N-2-Ferroceneethyl maleamic acid. Maleic anhydride (0.6637 g, 6.768 mmoles, 1.25eq) in dry THF (25 mL) was added dropwise to a solution of crude 2-ferroceneethylamine (1.2445 g, 5.428 mmoles, 1eq) in dry THF (50 mL). The mixture was chilled with an ice-water bath and stirred under nitrogen for 3 hours before the solvent was removed under vacuum. The crude product was purified by flash chromatography (1 % acetic acid, 20 % ethylacetate in dichloromethane) to give 1.595 grams (75 %) as a crystalline orange solid. *^{1}H NMR ($CDCl_3$/CD_3OD, δ ppm):* 6.39 (1H, d, J= 12.8 Hz, H_4); 6.24 (1H, d, J= 12.8 Hz, H_5); 4.43 (2H, br.s, NH, OH); 4.15 (5H, s, Ferrocenyl); 4.14 (2H, s, Ferrocenyl); 4.11 (2H, s, Ferrocenyl); 3.44 (2H, t, J= 7.4 Hz, H_1); 2.57 (2H, t, J= 7.4 Hz, H_2).

2-Ferroceneethyl Maleimide. Ammonium acetate (7.5 grams) was dissolved in 15 mL of acetic anhydride and heated to 80 °C under a nitrogen atmosphere. *N*-2-Ferroceneethyl maleamic acid (518.6 mg, 1.585 mmoles) in acetic anhydride (35 mL) was added over 3.5 hours and the

reaction was heated and stirred for a further 2.5 hours. The mixture was subsequently chilled in an ice-water bath and then added to an ice-water slurry (1.75 L). The aqueous mixture was extracted with ethyl acetate (5 x 150 mL) before the combined organics were washed with water (4 x 200 mL) then brine (3 x 50 mL), dried ($NaSO_4$) and concentrated en vacuo. Purification by flash chromatography (gradient of 5 % to 50 % ethyl acetate in hexane) gave 200.9 mg (41 %) of *N*-2-ferroceneethyl maleimide. *^1H NMR ($CDCl_3$, δ ppm):* 6.67 (2H, s, $H_{4,5}$); 4.16 (5H, s, Ferrocenyl); 4.10 (4H, s, Ferrocenyl); 3.64 (2H, s, H_1); 2.56 (2H, s, H_2).

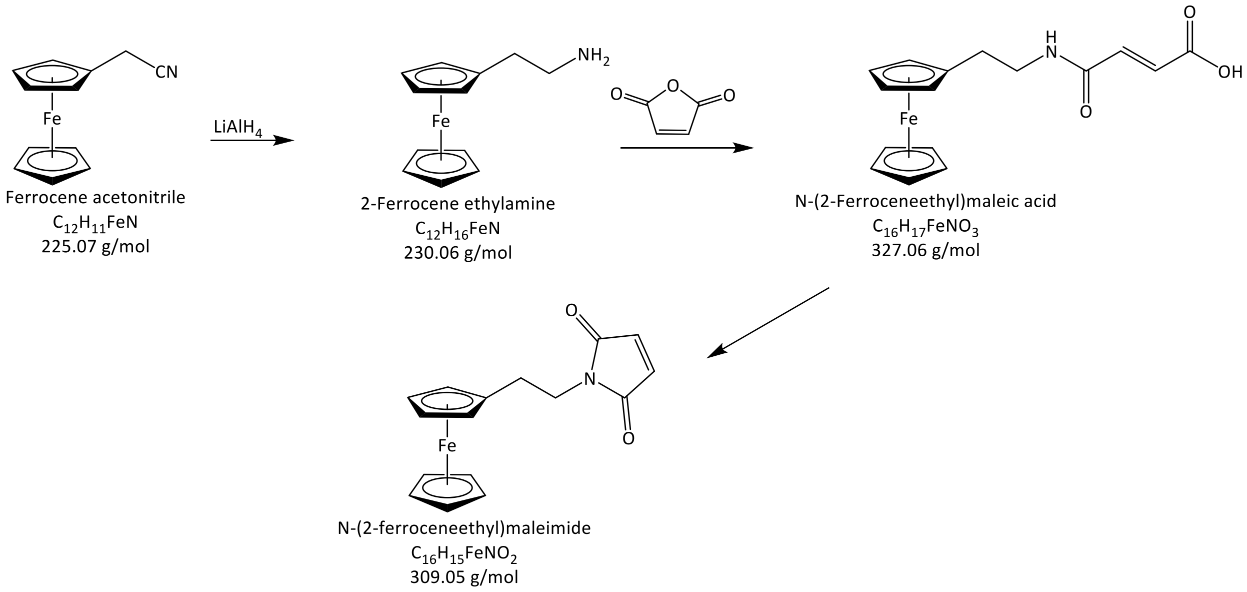

<u>Figure S1</u>. Synthesis of *N*-(2-ferroceneethyl) maleimide (FEM).

S1.2 Synthesis of ferrocenoylethylmaleimide (FMEA)

Ferrocenyl acid chloride. Ferrocene carboxylic acid (2-maleimidoyl)ethylamide merrocenoylethylmaleimide (FMEA) was synthesized as described by Siewert and Karst [14]. Briefly, ferrocene carboxylic acid (2.011 g, 8.74 mmol) was suspended in dry dichloromethane (100 mL) with 2-3 drops of dimethylformamide (DMF). Oxalyl chloride (2.2 mL, 26.0 mmol) was added dropwise over 5 minutes. On addition of the oxalyl chloride, the orange/brown

suspension turned to a dark red solution with some slight bubbling. After 40 minutes the reaction mixture was concentrated in vacuo to provide ferrocenyl acid chloride as a dark red solid that was used crude in the next step.

Ferrocenoylethylmaleimide (FMEA). *N*-(2-Aminoethyl)maleimide trifluoroacetate salt (1.008 g, 3.97 mmol) was dissolved in dry dichloromethane (55 mL), followed by the addition of triethylamine (2.3 mL, 16.5 mmol). To this was added a solution of ferrocenyl chloride (0.870 g, 3.50 mmol) in dry dichloromethane (60 mL), where a white gas was evolved upon mixing. After 55 minutes, the reaction mixture was washed with 0.1 M hydrochloric acid (2 x 70 mL), then water (2 x 70mL), dried over magnesium sulfate and concentrated to give an orange-brown solid (0.5150g, 42 % yield) that was confirmed to be FMEA by proton NMR. ^{1}H NMR (400 MHz, $CDCl_3$, δ ppm): 3.60 (2H, m), 3.80 (2H, m), 4.18 (5H, s), 4.33 (2H, t, J = 1.9 Hz), 4.65 (2H, t, J = 1.9 Hz), 6.76 (2H, s).

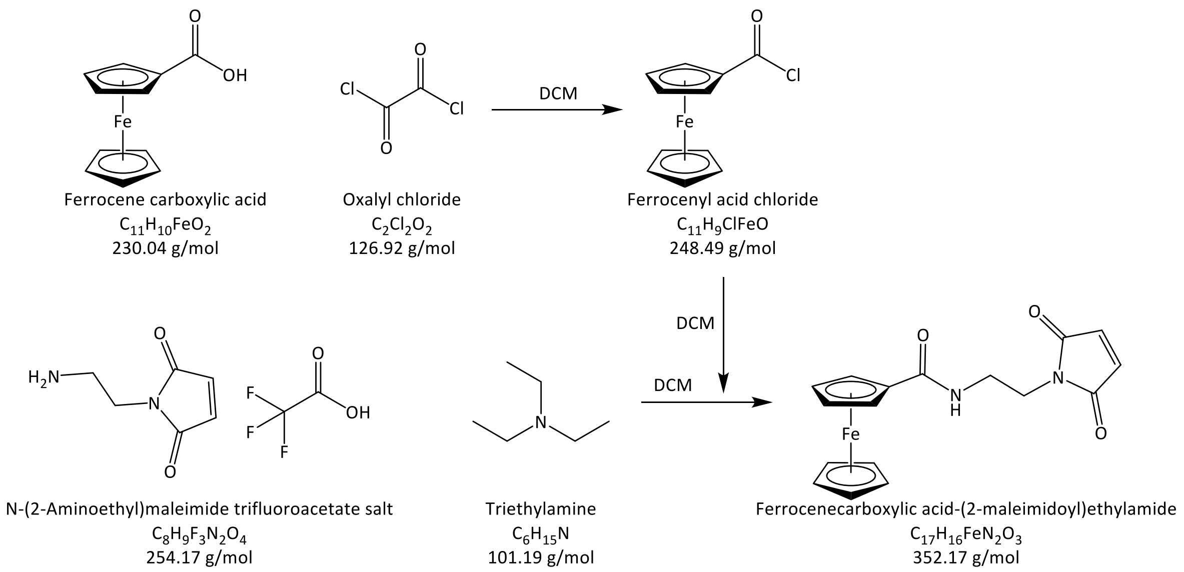

Figure S2. Synthesis of ferrocene carboxylic acid-(2-maleimidoyl)ethylamide (FMEA).

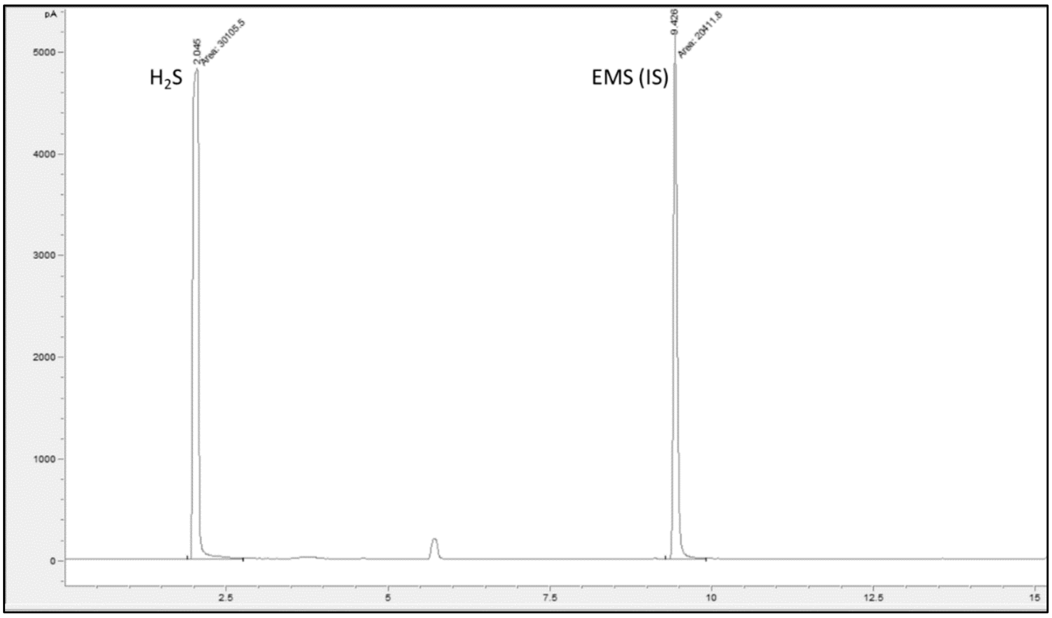

<u>Figure S3</u>. Liberation of hydrogen sulfide (H_2S) from 4-acetaminothiophenol (AATP) standard (3.0 g/L) prepared in Buffer A.

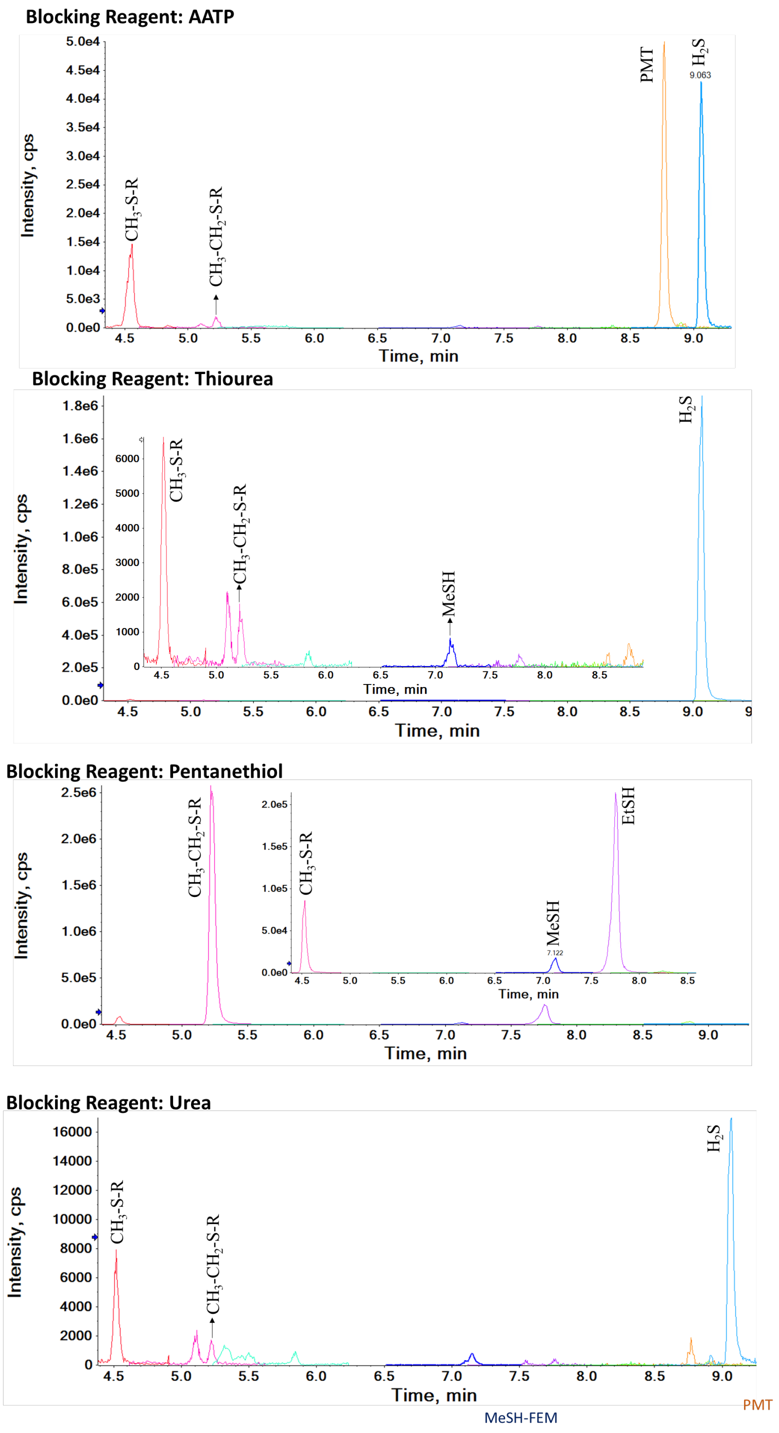

<u>Figure S4</u>. Comparison of interferences of four blocking reagents including AATP, thiourea, 1-pentanethiol and urea with the quantitation of target sulfhydryls in model wine solution.

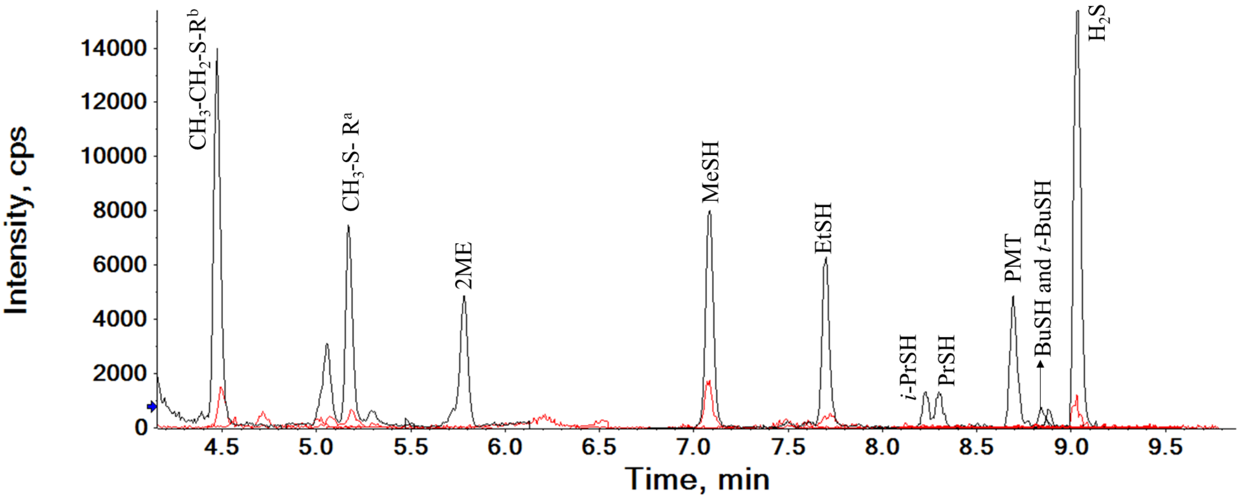

Figure S5. Selectivity in model wine solution. Chromatogram of derivatised blank model wine solution (red) and chromatogram of derivatised blank model wine containing CH_3-S-R species, CH_3-CH_2-S-R species, 2-mercaptoethanol (2ME), methanethiol (MeSH), ethanethiol (EtSH), *iso*-propanethiol (*i*-PrSH), propanethiol (PrSH), phenylmethanethiol (PMT), butylthiol (BuSH), *tert*-butylthiol (*t*-BuSH), and hydrogen sulfide (H_2S) at their LOQ levels (black).[a] is terminal methyl and R represents an organic substituent with a sulfur bridging atom and [b] is terminal ethyl and R represents an organic substituent with a sulfur bridging atom.

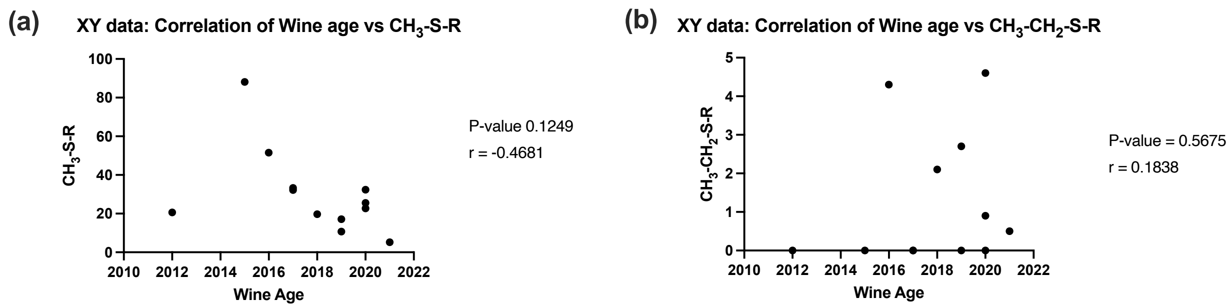

Figure S6. Pearson correlation between wine age and the concentrations (μg/L) of (a) TCEP-releasable CH_3-S-R species, and (b) TCEP-releasable CH_3-CH_2-S-R species measured in seven commercial white wines and seven commercial red wines.

Table S1. Comparison of peak area response for sulfhydryls and TCEP-releasable CH_3-S-R and CH_3-CH_2-S-R species in model wine by HPLC-MS/MS after derivatisation with FEM and FMEA.

Sample	MeSH	EtSH	CH_3-S-R[a]	CH_3-CH_2-S-R[a]	PMT	2ME	*i*-PrSH	PrSH	BuSH	H_2S
pH 3	245	92	11190	1758	3295	238	2891	463	330	245
pH 7	9856	3986	19822	16350	20524	12002	15380	13528	17915	9856

[a] Terminal methyl and R represents an organic substituent with a sulfur bridging atom.

Table S2. Intra- and inter-day repeatability for the analysis of sulfhydryls and TCEP-releasable CH_3-S-R species in white and red wine matrices by HPLC-MS/MS after derivatisation with FEM and FMEA.

Analyte	Model Wine	White Wine	Red Wine	Model Wine	White Wine	Red Wine
	Intra-day (n = 20) CV (%)			Inter-day (n = 6) CV (%)		
d3-CH_3-S-R[a]	7.4	7.3	9.6	8.0	5.7	6.4
d6-EtSH	9.8	4.9	5.9	6.5	4.4	5.7
d5-PMT	8.6	9.7	9.1	7.2	6.0	6.2

[a] Terminal methyl and R represents an organic substituent with a sulfur bridging atom.
